# The bio-adsorptive treatment of detergent: performance and mechanisms

**DOI:** 10.1038/s41598-026-59039-z

**Published:** 2026-06-30

**Authors:** Aya H. Saqr, Abd El-Latif Hesham, Mohamed I. Attia, Mohamed Abdel Rafea, Mahmoud A. Roshdy, Mohamed R. El-Aassar, Khalil I. Zarea, Mohammed N. Althuqbi, F. M. Mohamed

**Affiliations:** 1 Water and Environment Department, Faculty of Earth Sciences, Beni-Suef University, Beni-Suef City, Egypt; 2 Genetics Department, Faculty of Agriculture, Beni-Suef University, Beni-Suef, Egypt; 3 Department of Chemistry, College of Science, Imam Mohammad Ibn Saud Islamic University (IMSIU), Riyadh, 11623 Saudi Arabia; 4 Department of Physics, College of Science, Imam Mohammad Ibn Saud Islamic University (IMSIU), Riyadh, 11623 Saudi Arabia; 5 Department of Chemistry, College of Science, Jouf University, Sakaka, 2014 Saudi Arabia; 6 Department of Physical Sciences, Chemistry Division, College of Science, Jazan University, P.O. Box. 114, 45142 Jazan, Kingdom of Saudi Arabia

**Keywords:** Surfactant degradation, *Serratia plymuthica*, Activated carbon, Wastewater treatment, Engineering, Environmental sciences

## Abstract

This study develops an optimized hybrid system to treat sodium dodecyl sulfate (SDS)-contaminated wastewater that synergistically integrates microbial biodegradation with physical adsorption within a Response Surface Methodology (RSM) framework. A highly effective SDS-degrading bacterium, *Serratia plymuthica* strain BSU-AH-03, was isolated from hydrocarbon-contaminated soil. Using a Box-Behnken Design (BBD), the optimal biodegradation conditions (pH 7.9, 20 °C, 300 mg L⁻¹ SDS) yielded a degradation efficiency of 90.46% (predicted 91.27%, R² = 0.981). Subsequently, natural anthracite coal was employed as an adsorbent to polish the effluent. The anthracite exhibited a high surface area (890.9 m² g⁻¹) and a heterogeneous micro-mesoporous structure. Batch adsorption experiments achieved a maximum SDS uptake capacity of 158.7 mg g⁻¹ and a near-complete removal efficiency of 99.58% at 318 K and pH 7. The adsorption process was endothermic (ΔH° = 58.4 kJ mol⁻¹), spontaneous (ΔG° from – 5.15 to -10.65 kJ mol⁻¹), and followed pseudo-second-order kinetics and the Langmuir isotherm, indicating chemisorption as the dominant mechanism. Mechanistic analysis revealed that SDS adsorption involves intra-particle pore diffusion, hydrophobic interactions, electrostatic forces, and hydrogen bonding, culminating in interfacial hemi-micellar aggregation. The synergistic combination of tailored biodegradation and advanced adsorption provides a highly efficient, statistically optimized strategy for the complete remediation of surfactant-laden industrial effluents.

## Introduction

Surfactants are a broad class of amphiphilic compounds extensively used in detergents, textile processing, petroleum recovery, food industries, mining, and wastewater treatment due to their excellent interfacial activity and solubilization properties. Global surfactant production currently exceeds 7 million tons annually, resulting in the continuous discharge of surfactant-containing effluents into aquatic environments. Among these compounds, anionic surfactants such as sodium dodecyl sulfate (SDS) and linear alkylbenzene sulfonates (LAS) are considered major environmental contaminants because of their persistence, foaming behavior, and toxic effects on aquatic organisms and microbial communities^1,2^. Surfactant concentrations in industrial and domestic wastewaters may vary from a few milligrams per liter to several thousand milligrams per liter depending on the source and industrial activity^3–5^.

The release of untreated surfactant-rich wastewater causes significant environmental concerns, including reduction of dissolved oxygen, inhibition of microbial activity, membrane damage in aquatic organisms, and deterioration of water quality. Previous studies have demonstrated that surfactants may negatively affect aquatic ecosystems even at relatively low concentrations^6^. Consequently, the development of efficient, economical, and environmentally sustainable remediation technologies for surfactant-contaminated wastewater remains an important research priority. Various physicochemical and biological technologies have been investigated for surfactant removal, including coagulation-flocculation^7^, membrane filtration^8^, advanced oxidation^9^, constructed wetlands^10^, biodegradation^11^, and adsorption processes^12^. Among these methods, biological degradation and adsorption are considered particularly attractive because of their operational simplicity, cost-effectiveness, and high removal efficiency. Biodegradation enables the transformation of surfactant molecules into simpler and less harmful intermediates through microbial metabolic activity, whereas adsorption provides rapid polishing of residual contaminants through surface interactions and pore diffusion mechanisms^13^.

Recent studies have further highlighted the growing interest in low-cost adsorbents and hybrid treatment technologies for wastewater remediation. For example, recent studies have successfully employed magnetic chitosan/calcium alginate double-network hydrogel beads to remove anionic surfactants, cationic surfactants, and organic pollutants from aqueous solutions, demonstrating the importance of adsorption-based treatment technologies in sustainable wastewater management^12^. Likewise, biological treatment coupled with adsorption processes has shown promising performance due to the complementary advantages of microbial degradation and surface sequestration mechanisms^14–16^.

Although biodegradation can achieve substantial surfactant removal, incomplete mineralization and the persistence of intermediate products may still limit the final effluent quality^17^. Therefore, integrating adsorption as a post-treatment polishing step represents an effective strategy to enhance overall remediation efficiency. Natural carbonaceous materials such as anthracite coal have attracted increasing attention as adsorbents because of their high surface area, porous structure, chemical stability, and low operational cost^18,19^. In addition, adsorption efficiency is strongly governed by multiple operational parameters, including pH, temperature, surfactant concentration, and adsorbent dosage, which necessitate systematic optimization approaches.

Traditional one-factor-at-a-time optimization methods are often inadequate for complex multivariable systems because they fail to evaluate interaction effects among operational parameters and require extensive experimental effort. Response Surface Methodology (RSM), particularly the Box–Behnken Design (BBD), provides an efficient statistical framework for modeling, optimization, and evaluation of nonlinear interactions among process variables while minimizing the number of experimental runs^20,21^. Several recent environmental studies have successfully employed RSM-based optimization for adsorption and biodegradation systems, confirming its effectiveness in improving pollutant removal efficiency and process reliability^22,23^.

Therefore, the major innovation of the present work lies in the synergistic integration of optimized microbial biodegradation with anthracite-based adsorption for the efficient treatment of SDS-contaminated wastewater using a statistically optimized hybrid remediation platform. Specifically, this study: (i) isolated and identified an efficient SDS-degrading bacterial strain from contaminated soil; (ii) optimized the biodegradation process using BBD under RSM; (iii) evaluated the adsorption performance of natural An toward residual SDS through equilibrium, kinetic, and thermodynamic analyses; and (iv) proposed the dominant adsorption and removal mechanisms governing the integrated biodegradation–adsorption system. The developed hybrid approach aims to provide a cost-effective, sustainable, and highly efficient treatment strategy for surfactant-contaminated industrial wastewater.

## Materials and methods

### Materials

Commercial-grade anthracite (An) was supplied by Matrouh Company for Water and Wastewater, Egypt, while sodium dodecyl sulfate (SDS, purity 99.5%) was obtained from Sigma-Aldrich Co. (St. Louis, MO, USA). Hydrochloric acid (HCl), sodium hydroxide (NaOH), and the mineral salts used for preparation of the minimal salt medium (MSM), including disodium hydrogen phosphate (Na₂HPO₄), potassium dihydrogen phosphate (KH₂PO₄), and ammonium sulfate ((NH₄)₂SO₄), were procured from Al-Naser Company for Intermediate Chemicals (Cairo, Egypt). General-purpose agar medium used for bacterial cultivation and isolation was prepared in the laboratory according to standard microbiological procedures.

### Sample collection

A soil sample was taken from an area close to a fuel station owned by Ragai Abdel Fattah (Beni-Suef City, Egypt) that was contaminated with organic matter and industrial detergents. Soil samples were collected from the top layer at the fuel station’s external border, which is open to the public. The non-destructive sampling did not disrupt the station’s functioning. The research was conducted solely for academic purposes. The purpose of the sampling was to identify microorganisms that could potentially be used in greywater treatment and that could use synthetic detergents as their only carbon source. Samples were collected from three separate sites located 30 cm apart from a depth of 5 to 15 cm. After combining the subsamples, about 2.5 kg of soil was produced, which was then completely homogenized.

### Experimental

Microbial cultivation commenced with the inoculation of a minimal salts medium (MSM) using contaminated soil. The medium was amended with sodium dodecyl sulfate (SDS) at a concentration of 2 g L⁻¹, establishing it as the exclusive carbon substrate. The incubation was carried out for seven days at 100 rpm with constant orbital agitation. To selectively enrich microbial populations capable of surfactant degradation, a three-week enrichment protocol using sequential subculturing was implemented. This involved periodic transfers into fresh minimal salts medium supplemented with 0.5 g of detergent. A predominant isolate, known as strain BSU-AH-03, was obtained by subsequent purification on general-purpose agar and used as the biological agent in a 6-liter bioreactor treatment system. Distilled water, MSM, SDS at 0.1 g L⁻¹, and 250 mL of the bacterial culture made up the reactor’s composition. Constant aeration and regulated heating kept the system going.

Following the biological treatment phase, the resultant effluent was sent to an adsorption procedure. 25 mL of the water sample was mixed with 20 mg of finely ground anthracite coal (particle size < 100 μm) for the adsorption step. To systematically examine the effects of important variables, namely pH, adsorbent dosage, initial surfactant concentration, temperature, and contact time, on the removal efficacy, this mixture was agitated at 200 rpm. The mixture was centrifuged following the agitation period. The residual surfactant concentration in the supernatant was then determined using a COD test to ascertain the adsorption efficiency and the capacity of the anthracite coal.

### Bacterial isolation

In order to isolate detergent-degrading bacteria, a soil sample was taken from a fuel station in the Beni-Suef governorate of Egypt. Because of its consistent contamination with surfactant-rich effluents from facility and vehicle washing, this location was carefully selected. A mineral basal salt medium, a defined growth solution with necessary nutrients but no organic carbon, was used in the isolation procedure. Sodium dodecyl sulfate (SDS) was used as the sole carbon and energy source. Only microorganisms possessing the enzymatic capacity to metabolize this common detergent surfactant would be able to multiply and establish colonies thanks to this selective enrichment technique.

#### Molecular identification of isolated bacteria

A fundamental method in molecular taxonomy, 16 S ribosomal RNA (16 S rRNA) gene sequence analysis, was used to identify the chosen bacterial isolate to the species level. In accordance with Hesham’s methodology^17^, the procedure began with the extraction of total genomic DNA from pure culture. Then, using the universal primer pair 27 F and 1492R, which target conserved regions flanking the gene as determined by^24^, the nearly full-length 16 S rRNA gene was amplified via Polymerase Chain Reaction (PCR). To guarantee ideal amplification, the PCR conditions, including specific thermal cycling parameters, were followed as stated in^25^. Once the isolate’s ~ 1.5 kb amplicon was confirmed, the PCR products were purified to eliminate reaction impurities. The ABI 3730 automated sequencer (Macrogen, Korea) was then used to sequence the purified DNA in both directions using the same primers. Overlapping reads from this bidirectional sequencing were essential for creating a precise, high-quality consensus sequence for the isolate.

#### Comparison of 16 S rRNA gene sequences with GenBank database

Bioinformatics software was used to process and align the 16 S rRNA gene sequences that were obtained for the bacterial isolate. These sequences were compared using the BLAST tool to reference sequences found in the GenBank database in order to ascertain their taxonomic identity. Molecular Evolutionary Genetics Analysis (MEGA) software, version 11.0, was used to reconstruct an evolutionary tree for a strong phylogenetic analysis. The tree was built using the Jukes-Cantor model to estimate genetic distances and the neighbor-joining algorithm. The three branches’ statistical confidence was assessed using a bootstrap analysis with 1,000 replications. The evolutionary relationships and exact taxonomic position of the exact taxonomic position of the selected bacterial strain was definitively established by this thorough methodology, which adhered to the established protocol of^26^.

#### GenBank nucleotide sequences submission

The nucleotide sequences of the 16 S rRNA gene for the bacterial isolate, BSU-AH-03, have been formally archived in the GenBank database. The main genetic sequence database maintained by the National Centre for Biotechnology Information (NCBI) is available at https://www.ncbi.nlm.nih.gov/genbank/. A distinct and permanent accession number was given to the submitted sequence; strain BSU-AH-03’s was PV345590.1. For verification or comparative genomic purposes, researchers from all over the world can access and examine the precise genetic data used in this study using this accession number, which act as a direct digital identifier^27^.

### Characterization

Using Fourier-transform infrared spectroscopy (Vertex 70, Bruker, Germany), the functional group composition of the anthracite (An) was determined. Spectral acquisition was carried out throughout the 400–4000 cm⁻¹ wavenumber range. X-ray diffraction analysis was used to determine the anthracite’s (An) mineralogical composition. A Shimadzu XRD-7000 Series instrument (Japan) set up with Cu Kα radiation (λ = 1.5419 Å) was used to obtain the diffractograms. With a step increment of 0.02° and a scanning rate of 0.5° per minute, the data collection covered a 2θ range of 5–50°. Field-emission scanning electron microscopy (FESEM) in conjunction with an Oxford energy-dispersive X-ray spectroscopy (EDS) system for elemental composition analysis was used to characterize the surface morphology of the synthesized materials. A BELSORP MINI X analyzer was used to apply the Brunauer-Emmett-Teller (BET) method to nitrogen physisorption isotherms measured at 77 K in order to determine specific surface area. Additionally, thermogravimetric analysis (TGA) was further used to assess the anthracite powder’s thermal degradation behavior. Thermogravimetric (TG) and derivative thermogravimetric (DTG) curves were recorded using a Shimadzu DT-50 thermobalance, by heating the specimens from 50 to 1000 °C at a rate of 10 °C min⁻¹ in a nitrogen atmosphere with a flow rate of 50 mL/min.

### Biodegradation optimization by BBD

A Box-Behnken Design (BBD) was used in this study to optimize the SDS biodegradation process.

**Table 1:** Factors and their ranges and levels for BBD.

Factor	Name	Units	Ranges	Minimum	Maximum	Mean
A	pH	-	7.4–7.9	7.4	7.9	7.65
B	Temperature	°C	15–25	15	25	20
C	SDS concentration	mg/L	300–600	300	600	450

The selected ranges for pH (7.4–7.9), temperature (15–25 °C), and initial SDS concentration (300–600 mg L⁻¹) were determined from preliminary one-factor-at-a-time experiments, which identified these intervals as the most effective for sustaining high SDS degradation activity by *Serratia plymuthica* BSU-AH-03. Outside these ranges, bacterial growth and degradation efficiency declined markedly due to pH stress, thermal inactivation, or substrate inhibition.

Therefore, in order to optimize the degradation efficiency, the experimental design comprised 17 runs, which methodically examined three operational parameters at three different levels, including five replicates of the central point. The three independent variables pH (A), temperature (B), and SDS concentration (C) that were determined to be crucial operational parameters based on preliminary research are shown in Table 1. SDS removal efficiency, the main response metric for ensuing biodegradation investigations, was used to quantify the impact of these variables. A second-order polynomial model was used to mathematically depict the functional relationship between the independent variables and the percentage of SDS degradation (Y)^28^.

1 $$Y = \beta o + \sum \beta _{i} X_{i} + \sum \beta _{{ii}} X_{i} ^{2} + \sum \sum \beta _{{ij}} X_{i} X_{j}$$

**Table 2:** BBD matrix observed removal rates and predicted values from BBD modeling.

Run	Factor 1	Factor 2	Factor 3	SDS degradation %	
	A: pH	B: Temperature (°C)	C: SDS conc. (mg/L)	Experimented Value (%)	BBD predicted
1	7.65	20	450	74.77	75.51
2	7.9	15	450	81.25	80.48
3	7.9	20	600	80.91	81.82
4	7.9	25	450	88.9	87.95
5	7.9	20	300	90.46	91.27
6	7.65	25	300	83.463	83.6
7	7.65	25	600	75.283	75.32
8	7.4	20	300	76.306	75.4
9	7.4	15	450	63.446	64.4
10	7.65	20	450	76.003	75.51
11	7.4	20	600	67.74	66.93
12	7.65	20	450	75.61	75.51
13	7.65	15	600	66.613	66.47
14	7.65	15	300	76.15	76.11
15	7.65	20	450	75.61	75.51
16	7.4	25	450	72.506	73.27
17	7.65	20	450	75.55	75.51

In the proposed statistical framework, the dependent variable (Y) represents the expected efficiency of SDS decomposition. The symbols X_i_ and X_j_ refer to the encoded independent variables, with βo functioning as the model’s constant term, while βi, βii, and βij denote the respective coefficients for linear, quadratic, and interactive effects. The removal efficiency (R%) was subsequently calculated using Eq. (3).

Using anthracite coal as the sorbent medium, the clarified supernatant from the optimized biodegradation process was then subjected to adsorption treatment.

This secondary treatment was carried out using a DOS-20 L orbital shaker system in batch mode. A series of SDS solutions made by methodically diluting the clarified post-biodegradation supernatant were used in the adsorption tests. 25 mL aliquots of each solution concentration were treated with precisely measured masses of an adsorbent, ranging from 2 to 40 mg. This study looked at SDS removal at specific operating temperatures (293–318 K), initial concentrations (5–120 ppm), and pH 3.0–10.0 (adjusted with 0.01 M NaOH/HCl). Comprehensive analysis of the effects of parameters on removal performance under controlled chemical conditions was enabled by this methodological framework.

The samples were shaken for 120 min in isothermal conditions as part of the adsorption tests. Using a HACH DRP 200 analyser, the subsequent SDS concentrations were measured as chemical oxygen demand (COD). Equations 2 and 3 were then used to calculate the adsorption capacity (qₑ) and contaminant removal efficiency.

2 $$\:{\mathrm{q}}_{\mathrm{e}}=\frac{({\mathrm{C}}_{0}-{\mathrm{C}}_{\mathrm{e}})}{\mathrm{M}}\times\:\mathrm{V}$$

3 $$\:\mathrm{R}=\frac{({\mathrm{C}}_{0}-{\mathrm{C}}_{\mathrm{e}})}{{\mathrm{C}}_{0}}\times\:100$$

M represents the mass of the adsorbent. C_o_ and C_e_ (both in mg/L) denote the initial and equilibrium SDS concentrations, respectively, while R indicates the removal percentage. V is the volume of the solution.

## Results and discussion

### Isolation and molecular identification of bacterial isolates

In this investigation, one bacterial isolate, designated BSU-AH-03, was isolated from a soil sample collected from a fuel station in Beni-Suef, Egypt, an environment hypothesized to be enriched with microorganisms capable of degrading hydrocarbon-based contaminants. Establishing the exact taxonomic identity of these isolates through genetic characterization was the main goal, as this is an essential first step for any biotechnological applications that follow. Comprehensive 16 S ribosomal RNA (16 S rRNA) gene sequencing, the basis of contemporary bacterial phylogenetic and identification, was used for this characterization.

The isolate had a clear taxonomic assignment based on the analytical results. In the GenBank database, the 16 S rRNA gene sequence of isolate BSU-AH-03 was identical to that of strains of another genus, Serratia plymuthica strain K-7 (NR_037111.1) and S. plymuthica strain NBRC 102,599 (NR_114158.1), according to comparative sequence analysis. Its identification as Serratia plymuthica strain BSU-AH-03, which has been archived under the accession number PV345590.1, was made possible by this clear genetic correspondence. To move beyond simple sequence similarity and robustly confirm the evolutionary relationships of this strain, detailed phylogenetic tree was reconstructed using the neighbor-joining algorithm.

**Fig. 1:**
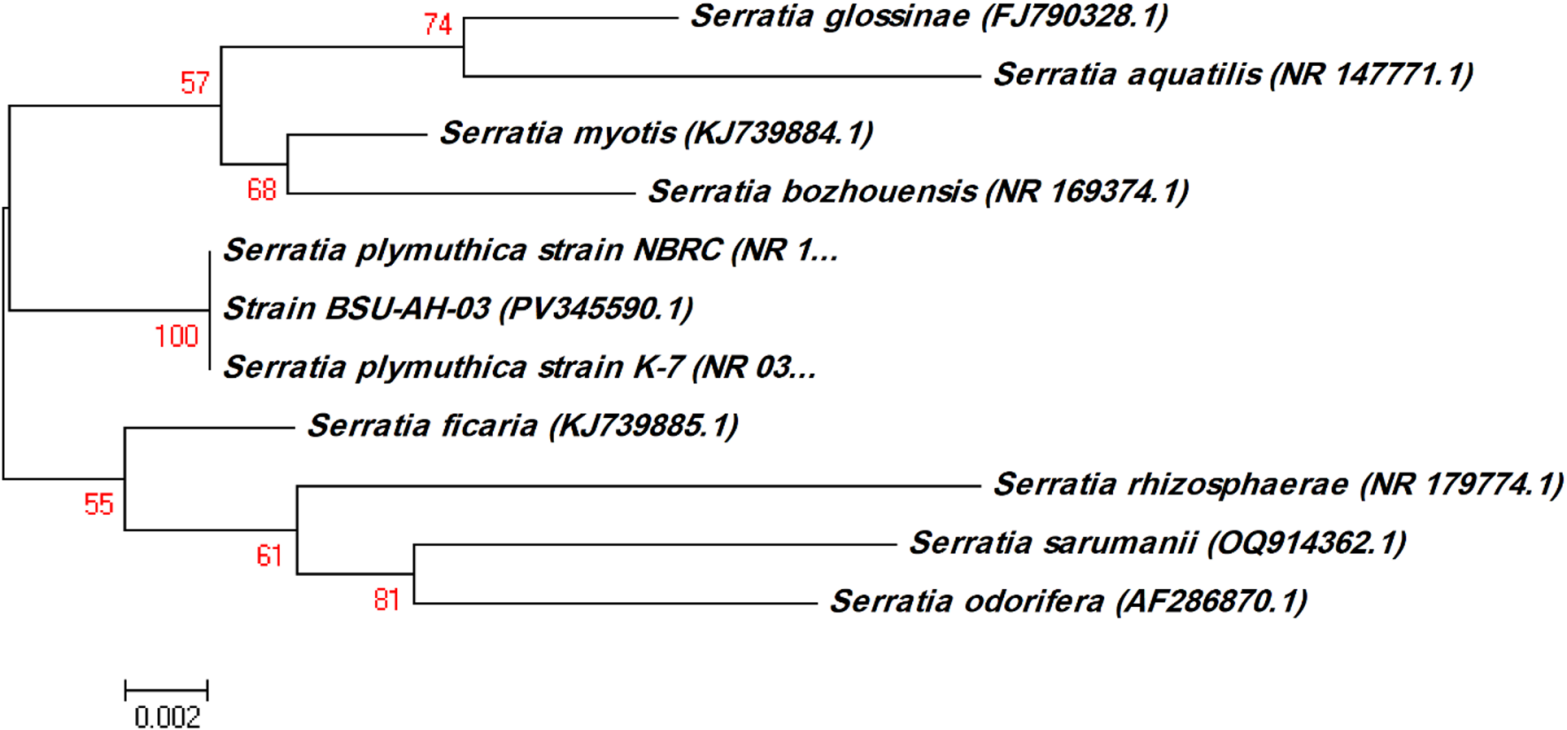
Phylogenetic relationships between strain BSU-AH-03 and 16 S rRNA gene sequences from other published Serratia spp. GenBank accession numbers are given in parentheses. The phylogenetic tree was constructed by the neighbor-joining method showing the position of strain BSU-AH-03.

Figure 1 clearly positions strain BSU-AH-03 within a well-supported cluster of Serratia species. These phylogenetic analyses provide a visual and statistical confirmation of the real taxonomic position of the strain, validating the identification obtained from the BLAST comparisons and solidifying the foundation for its recognition as a detergent-degrading member of the genus *Serratia*.

### Characterization

#### Infrared spectroscopy

**Fig. 2:**
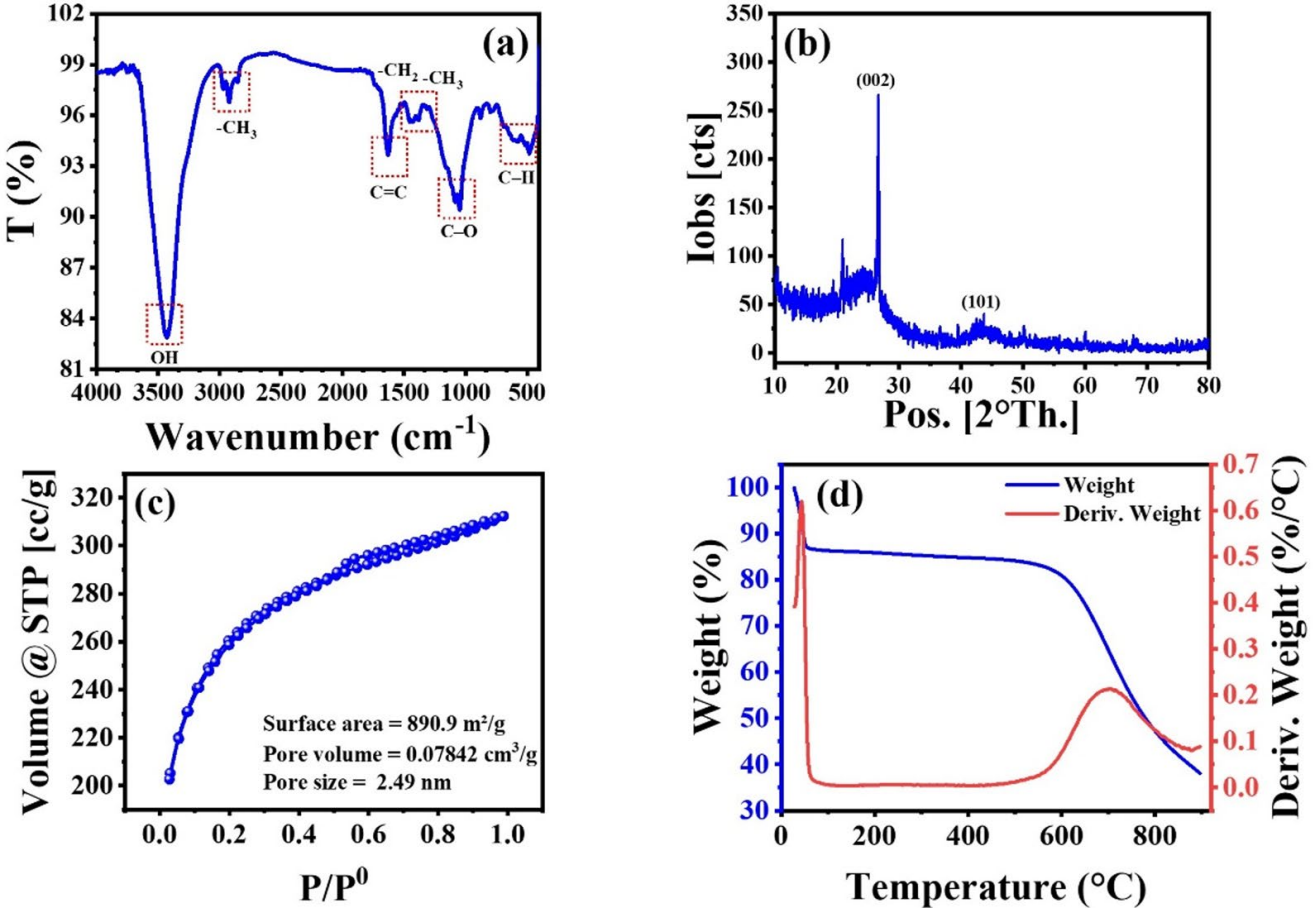
(**a**) FTIR spectrum, (**b**) XRD and (**c**) BET studies, and (**d**) TGA.

The FTIR spectrum of the anthracite coal powder is presented in Fig. 2a, revealing characteristic absorption bands that delineate four principal spectral domains: the aromatic region (700–900 cm⁻¹), the oxygen-containing functional group region (1000–1800 cm⁻¹), the aliphatic region (2800–3000 cm⁻¹), and the hydroxyl stretching region (3000–3600 cm⁻¹)^29^. Specific vibrational modes were identified, including aromatic C–H deformation within the 700–900 cm⁻¹ range and C–O stretching vibrations of phenolic, alcoholic, etheric, and lipidic structures at 1085 cm⁻¹. The bending vibrations of the methyl (-CH₃) and methylene (-CH₂-) groups were identified as two distinct peaks at 1381 and 1455 cm⁻¹, respectively^30^. The absorption at 2918 cm⁻¹ suggested asymmetric -CH₃ stretching, whereas a band at 1631 cm⁻¹ was ascribed to C = C aromatic ring stretching. Additionally, the presence of hydroxyl groups involved in intermolecular hydrogen bonding was indicated by a broad peak at 3428 cm⁻¹^18,31,32^.

#### XRD spectroscopy

The multiphase composition of the raw anthracite sample, which includes both crystalline and amorphous components, was confirmed by X-ray diffraction (XRD) analysis (Fig. 2b). The noticeable broad hump with centered at 20–30° (2θ) is indicative of a primarily amorphous carbon structure, which is caused by disordered, non-graphitic carbon arrangements. Additionally, distinct crystalline reflections that are indexed to the (002) basal plane of graphitic carbon were observed at a 2θ position of 26.4° (JCPDS 41-1487). The coexistence of ordered graphitic crystallites within the primarily amorphous carbon matrix is confirmed by this discovery. Furthermore, the structural order associated with aromatic ring condensation is characterized by a diffraction feature found at roughly 43°, which corresponds to the (10 L) plane. This signal provides important information about the aromatic layer assemblies’ alignment and structural integrity^33,34^.

#### BET studies

The anthracite sample’s textural characteristics, such as its specific surface area, pore volume, and pore size distribution, were characterized using nitrogen physisorption measurements (Fig. 2c). According to IUPAC guidelines, the resulting adsorption-desorption isotherm corresponded to a Type IV isotherm with a corresponding H4 hysteresis loop. In line with previously reported results, the pore size distribution, which was obtained from the derivative dV/d(log D), showed a heterogeneous porosity profile across several diameter ranges. With a mean pore diameter of 2.49 nm and a BET surface area of 890.9 m²/g, the textural parameters displayed in Fig. 2c confirmed the microporous structure of An. Faster adsorption kinetics are encouraged by a large surface area because it lowers mass transfer resistance. In order to ensure high removal efficiency and quick uptake rates, which will result in better performance in the remediation of surfactant-contaminated effluents, it is crucial to maximise the anthracite’s surface area^35^.

#### Thermogravimetric analysis (TGA)

As shown in Fig. 2d, thermogravimetric analysis (TGA) and its derivative (DTG) were used to evaluate the thermal stability of the anthracite (An). A clear decomposition pattern is shown by the profiles, which begin with a small mass loss (< 5%) below 100 °C due to the development of physisorbed surface moisture. Between 100 °C and 450 °C, there was a subsequent mass reduction of about 15%, which is suggestive of progressive thermal decomposition processes. The An adsorbent showed a more significant mass reduction, primarily due to the thermal breakdown of persistent organic matter. Following thermal treatment at 600 °C, which is marked by an abrupt decrease in mass, the sample maintained a residual mass fraction of about 40%^36^.

#### Mineralogy investigation

SEM analysis was used for morphological characterization, to evaluate the texture, homogeneity, and particle distribution.

**Fig. 3:**
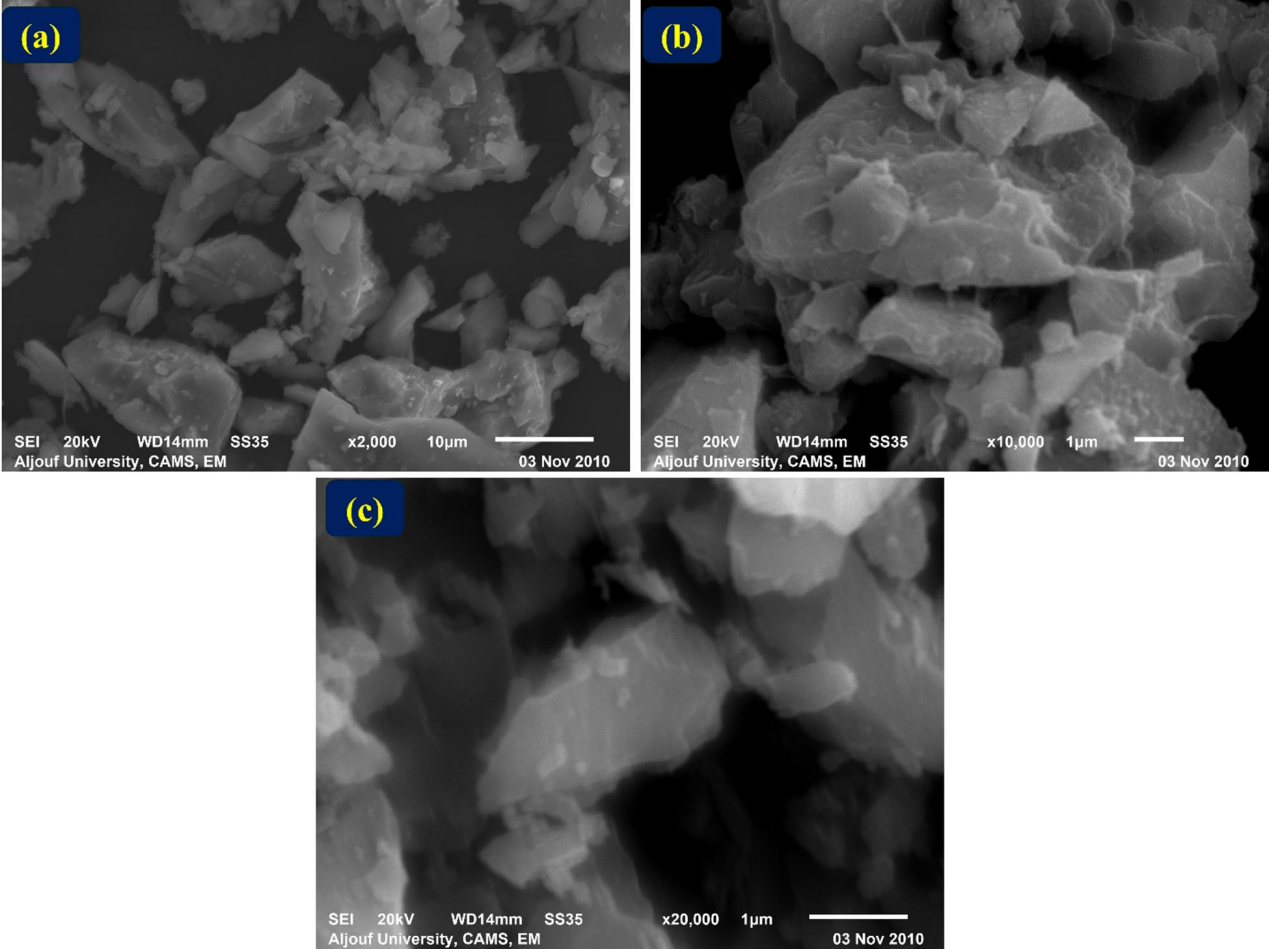
Microstructural and compositional analysis of the An via FESEM imaging.

The material’s advanced metamorphic grade and significant carbonaceous composition are consistent with the representative images in Fig. 3, demonstrating a densely aggregated architecture. Rough, fractured areas were observed as well as smooth, vitrified sections on the surface, giving it an uneven texture.

These structural characteristics indicate a friable coal matrix that formed during the coalification process under extreme thermo-metamorphic conditions. A heterogeneous array of micropores and mesopores dispersed throughout the surface is also revealed by microscopic analysis. The majority of the pore architecture is made up of slit-shaped, anisotropic voids with limited connectivity. Anthracite differs from its sub-bituminous counterparts in that it typically has a lower macroporous content but still has an advanced microporous structure that aids in the removal of adsorptive surfactants.

### SDS degradation and optimization using BBD

The BBD was employed to optimize the biodegradation of SDS by evaluating the effects of three independent operational variables, namely pH, temperature, and initial SDS concentration. The selected experimental domain and variable ranges are summarized in Table 1. The experimental matrix consisted of 17 runs, including five replicated center points to estimate the pure experimental error and improve the statistical reliability of the model. The experimentally observed and model-predicted biodegradation efficiencies are presented in Table 2. The response data obtained were fitted to a second-order polynomial model expressed as:

4 $$Y = 75.51 + 7.69A + 4.09{\text{ }}B - 4.48{\text{ }}C{-}0.352AB{-}0.245{\text{ }}AC + 0.340{\text{ }}BC + 2.24{\text{ }}A^{2} {-}1.23{\text{ }}B^{2} + 1.10{\text{ }}C^{2}$$

where Y represents the SDS degradation efficiency (%), while A, B, and C correspond to pH, temperature (°C), and SDS concentration (mg L⁻¹), respectively.

ANOVA demonstrated that the developed quadratic model was highly significant, with an F-value of 91.63 and a probability value below 0.0001, confirming the statistical significance of the regression equation. The linear effects of pH, temperature, and SDS concentration were all statistically significant (*p* < 0.0001), indicating that each parameter independently exerted a substantial influence on the biodegradation process^37^. Significant quadratic contributions were also observed for the pH and temperature terms, as evidenced by the significance of A^2^ (*p* = 0.0023) and B^2^ (*p* = 0.0374), revealing the presence of curvature in the response surface. In contrast, the quadratic effect of SDS concentration was statistically insignificant (*p* = 0.0562). Furthermore, the interaction terms among the investigated variables (AB, AC, and BC) were not significant (*p* > 0.05), suggesting that the operational factors influenced SDS biodegradation in an essentially additive manner within the investigated ranges, thereby simplifying process optimization and operational control^38^.

The adequacy and predictive capability of the developed model were confirmed through multiple statistical indicators. The coefficient of determination demonstrated that the model explained approximately 98.1% of the total variability in the experimental data (R^2^ = 0.981). The close agreement between the adjusted R^2^ (R_Adj_^2^ = 0.960) and predicted R^2^ (R_Pred_^2^ = 0.879) with a difference of only 0.081 indicated the absence of substantial overfitting and confirmed satisfactory predictive consistency^35^. Additionally, the adequate precision ratio reached 35.56, which greatly exceeded the minimum desirable value of 4, demonstrating a strong signal-to-noise ratio and confirming that the model could effectively navigate the design space. The low coefficient of variation (CV = 1.29%) reflected excellent reproducibility and experimental precision, while the relatively small PRESS value (97.04) compared with the total sum of squares (807.36) further supported the robustness and predictive reliability of the statistical model^20^.

**Fig. 4:**
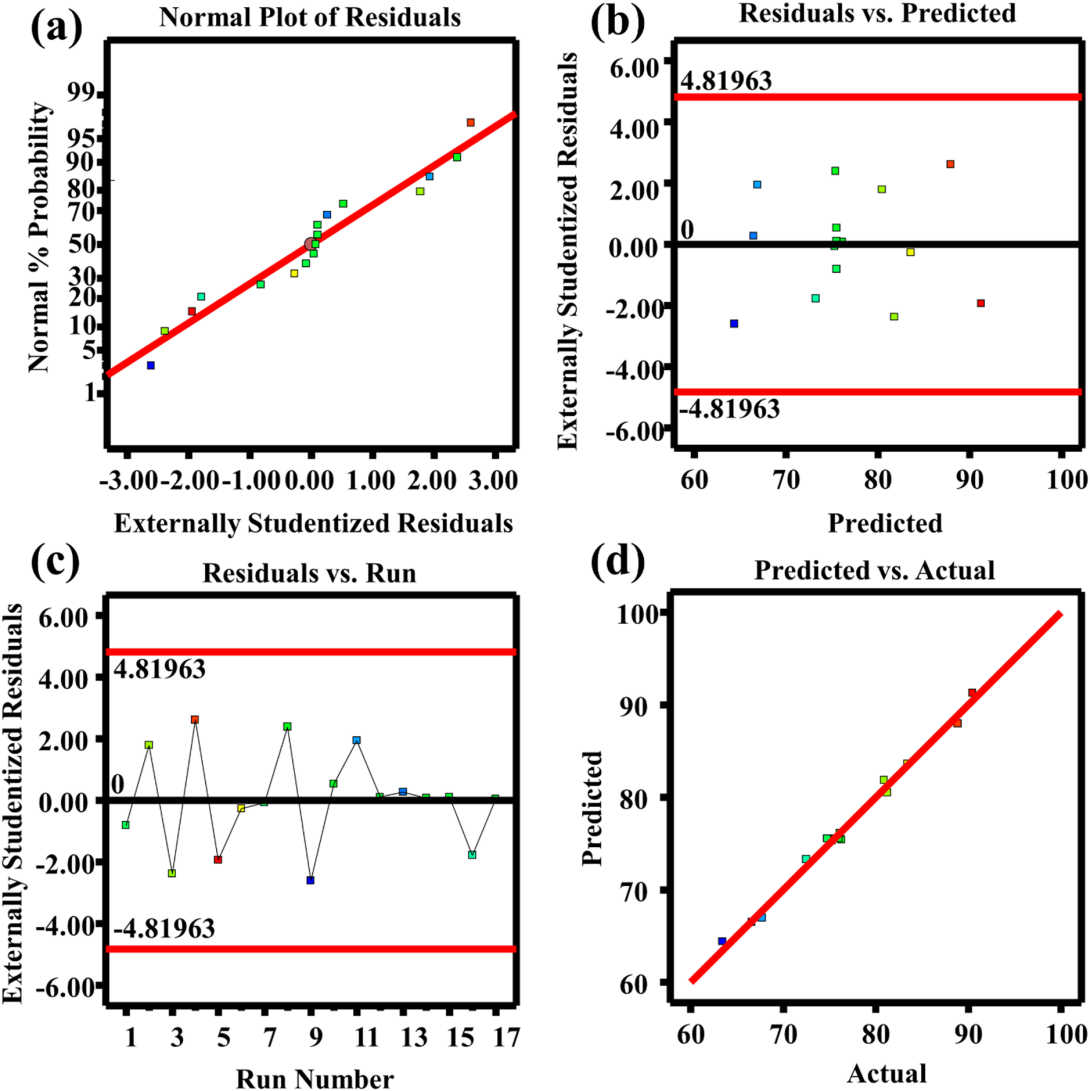
Diagnostic plots for model validation: (**a**) normal probability distribution of residuals; (**b**) residuals as a function of predicted values; (**c**) residual variation across experimental sequence; and (**d**) correlation between predicted and observed responses.

A thorough diagnostic evaluation of the proposed model was conducted by inspecting the residual distributions graphically. As shown in Fig. 4a, the normal probability plot served to assess the residual pattern a fundamental diagnostic tool for verifying conformity with the underlying statistical assumptions. The random alignment of data points along the 45° reference line provides compelling visual evidence of correct model specification, thereby reinforcing the inferential conclusions derived from the ANOVA. The appropriateness of the constructed model was further appraised using externally studentized residuals plotted against the predicted values (Fig. 4b). The data points exhibit a close approximation to the line of best fit, which is evident from the plot of actual versus forecasted values, indicating a robust concordance between experimental measurements and model predictions. In addition, a residual sequence plotted against the experimental run order (Fig. 4c) was examined to confirm the independence of error terms throughout the removal process. The scattered, non-systematic distribution of residuals around zero in Fig. 4c further supports the predictive reliability of the model. Finally, as illustrated in Fig. 4d, a strong linear relationship between the experimentally observed decolorization efficiencies and those predicted by the model underscores both the practical applicability and the predictive veracity of the developed framework^21^.

Experimental validation of the optimized conditions further verified the predictive capability of the BBD model. Validation experiments performed at the model-predicted optimum conditions (pH 7.9, 25 °C, and 300 mg L⁻¹ SDS) produced an average biodegradation efficiency of 90.46 ± 1.82%, which closely matched the predicted value of 91.27%, corresponding to a relative error of only 0.89%. Additional validation experiments conducted under randomly selected operational conditions also demonstrated excellent agreement between experimental and predicted values. At pH 7.65, 20 °C, and 450 mg L⁻¹ SDS, the experimentally obtained degradation efficiency was 75.55 ± 1.21% compared with a predicted value of 75.51%, while at pH 7.4, 20 °C, and 600 mg L⁻¹ SDS, the experimental efficiency reached 67.74 ± 1.45% versus a predicted value of 66.93%. All validation results fell within the 95% prediction intervals, thereby confirming the reliability and practical applicability of the developed optimization model.

**Table 3:** ANOVA table for SDS degradation by BBD model.

Source	Sum of Squares	df	Mean Square	F-value	*p*-value	
Model	800.57	9	88.95	91.63	< 0.0001	Significant
A-pH	473.15	1	473.15	487.39	< 0.0001	Significant
B-Temperature	133.58	1	133.58	137.6	< 0.0001	Significant
C-Initial concentration	160.56	1	160.56	165.39	< 0.0001	Significant
AB	0.497	1	0.497	0.512	0.4974	Not significant
AC	0.2401	1	0.2401	0.2473	0.6342	Not significant
BC	0.4624	1	0.4624	0.4763	0.5123	Not significant
A²	21.22	1	21.22	21.85	0.0023	Significant
B²	6.37	1	6.37	6.56	0.0374	Significant
C²	5.07	1	5.07	5.22	0.0562	Not significant
Residual	6.8	7	0.9708			
Lack of Fit	5.99	3	2	9.86	0.0255	significant
Pure Error	0.8093	4	0.2023			
Cor Total	807.36	16				
R^2^	0.981	Ad. precision	35.56			
R_Adj_^2^	0.960	CV%	1.29			
R_Pred_^2^	0.879	PRESS	97.04			

Despite the excellent statistical performance of the model, the lack-of-fit test was found to be statistically significant (*p* = 0.0255), indicating that the quadratic equation may not perfectly represent the true response behavior across all regions of the experimental domain.

**Fig. 5:**
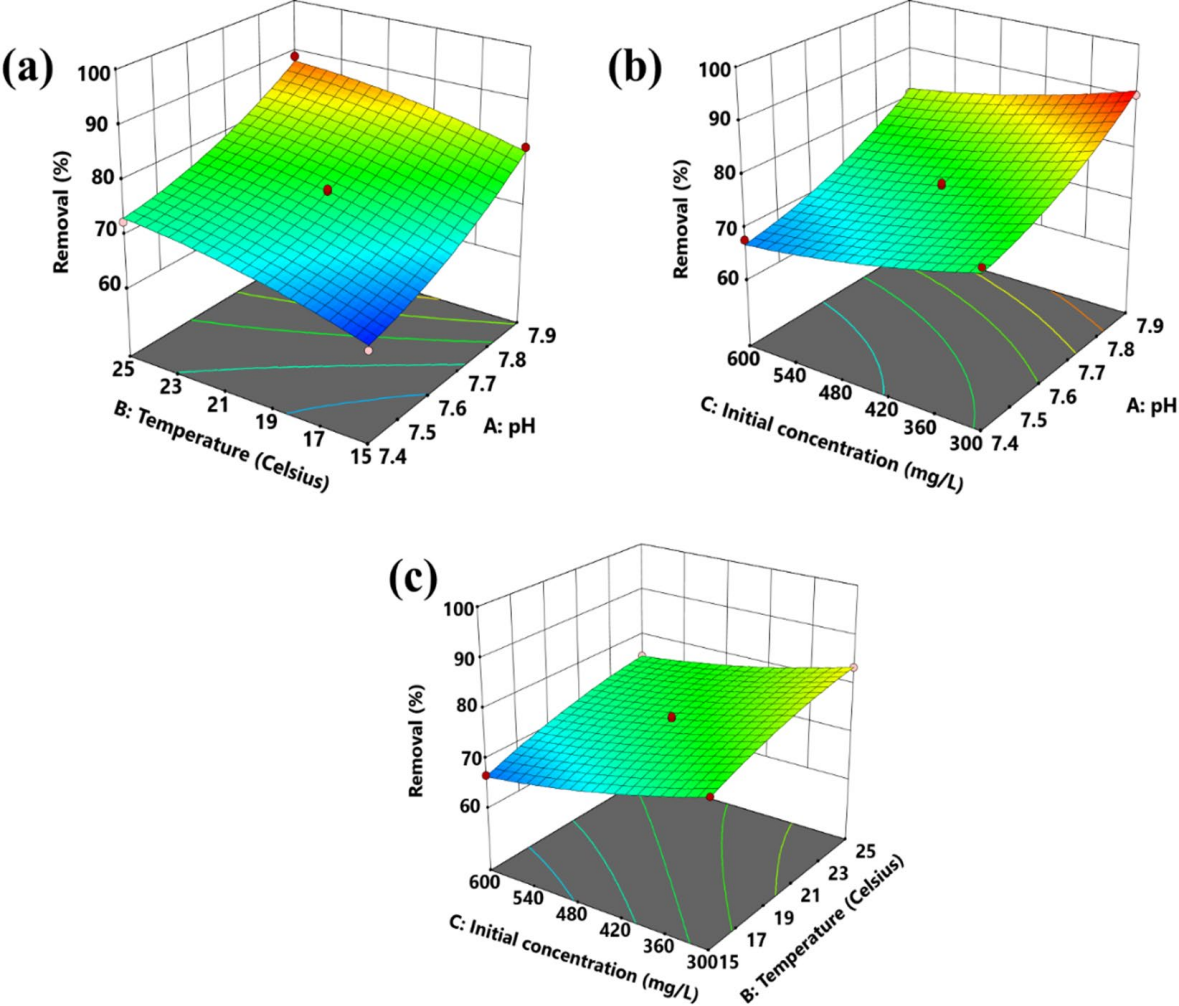
Response surface plots of the interactions between pH and temperature (**a**), pH and SDS concentration (**b**), and SDS concentration and temperature (**c**).

Nevertheless, the observed deviation was relatively minor, as reflected by the very low pure error value (0.2023), suggesting that the practical influence of this lack of fit remained limited. Moreover, the current model was established using a single bacterial isolate under controlled laboratory conditions; consequently, the application of the model to mixed microbial consortia or real wastewater matrices may introduce additional sources of variability not fully captured within the present investigation^39,40^.

Three-dimensional surface plots in Fig. 5 illustrate how the operational parameters interact to affect the system’s response. With the third variable held constant, these graphical representations show how two independent variables work together to affect the final output^41^.

The synergistic effects of temperature and pH on SDS degradation efficiency at a fixed surfactant concentration of 500 mg/L are shown in Fig. 5(a). According to the analysis, moderately acidic/alkaline conditions along with high temperatures are ideal for removal. As shown in Table 3, statistical analysis verified that the individual main effects of pH and temperature were highly significant (*p* < 0.0001), whereas their interaction (AB) was not significant (*p* = 0.4974).

Figure 5(b) shows the synergistic effect of SDS concentration and solution pH on degradation efficiency at a fixed temperature. According to the response surface analysis, near-neutral pH and low surfactant concentration lead to the best removal results. A highly significant interaction (*p* < 0.0001) between these two parameters is confirmed by statistical analysis (Table 3) confirms that the individual main effects of pH and SDS concentration were highly significant (*p* < 0.0001), but their interaction (AC) was not significant (*p* = 0.6342)^1^.

The combined effects of temperature and SDS concentration on performance degradation under fixed pH conditions are shown in Fig. 5(c). High temperatures and low starting surfactant concentrations produced the best efficiency. The individual main effects of temperature and SDS concentration were highly significant (*p* < 0.0001), but their interaction (BC) was not significant (*p* = 0.5123), as shown in Table 3. Temperature and concentration have a strong correlation, as indicated by the significant p-value (< 0.0001). The main removal pathway was determined to be biological degradation after additional control tests ruled out significant adsorptive losses to bacterial biomass^42^.

### Adsorption studies

#### Influence of solution pH

**Fig. 6:**
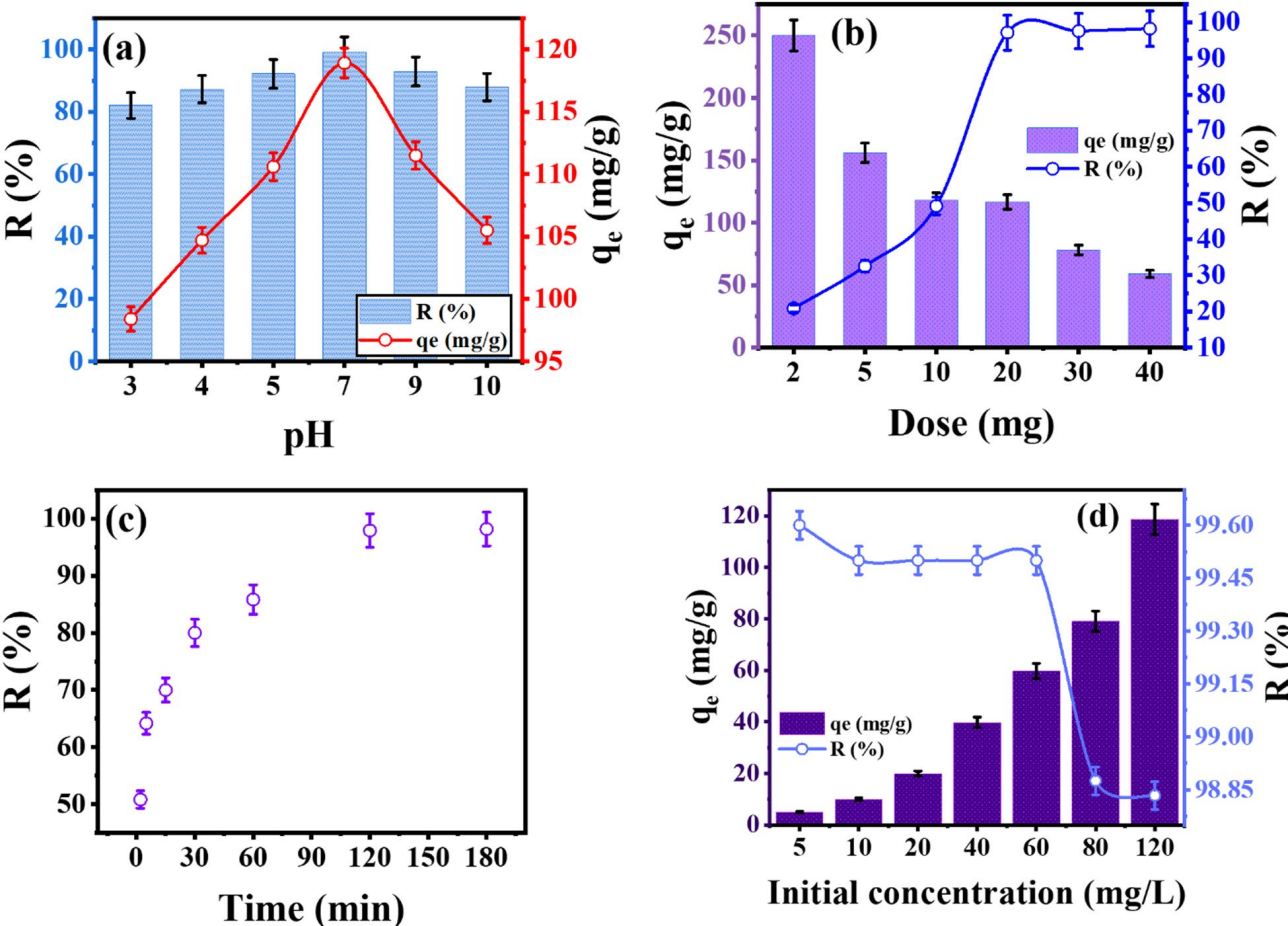
The effect of pH (**a**), effect of dosage (**b**), contact time (**c**), and SDS concentration (**d**) on the adsorption of SDS onto An.

A series of batch adsorption experiments were carried out at various pH levels to assess the impact of pH on the adsorption of sodium dodecyl sulphate (SDS). The adsorption efficiency of SDS decreases with increasing pH, as shown in Fig. 6a. Nonetheless, a clear pattern can be seen in the equilibrium adsorption capacity, which rises gradually as pH rises from 3 to 7 before steadily declining as pH rises from 7 to 10. As a result, the ideal pH for An’s uptake of SDS was determined to be 7, which translates to a maximum uptake of 118.9 ± 5 mg/g.

Furthermore, the point of zero charge (pH_PZC_) is a crucial factor that controls an adsorbent’s surface charge density and, as a result, affects its adsorption efficiency by altering the electrostatic interactions between the adsorbent and the adsorbate, thus the pH_PZC_ of the An was determined to be 7.52. Below this pH, the surface carries a net positive charge due to protonation of oxygen-containing functional groups (e.g., –OH₂⁺, –COOH₂⁺); above pHpzc, deprotonation leads to a net negative surface charge (e.g., –O⁻, –COO⁻)^43^. So that, the neutral protonated form of SDS predominates in acidic environments, greatly lowering its degree of dissociation and, as a result, diminishing its electrostatic attraction to the charged An surface. On the other hand, because of its intrinsic anionic surfactant characteristics, SDS completely dissociates in alkaline environments, producing a hydrophilic headgroup that is negatively charged.

The pH-dependent behavior of the adsorption system is characterized by clear mechanistic constraints. Strong electrostatic repulsive forces between the negatively charged adsorbent surface and anionic adsorbate species significantly reduce SDS adsorption capacity in alkaline environments. On the other hand, adsorption performance is limited by acidic media due to a combination of reduced structural stability of the adsorbent and inadequate SDS dissociation. Neutral pH (7.0), which represents an ideal balance between these conflicting pH-dependent effects, is where maximum uptake efficiency occurs^44^.

More specifically, to quantify the electrostatic contribution to SDS adsorption, the surface potential (ψ₀, in mV) was estimated using the Nernst-type equation commonly applied to amphoteric surfaces^45^:

$$\psi _{o} {\text{ }} = 2.303\left( {RT/F} \right)\left( {pHpzc{-}pH} \right)$$

where *R* = 8.314 J mol⁻¹ K⁻¹, T = 298 K, and F = 96,485 C mol⁻¹.

Therefore, at pH 3.0, this gave ψ₀ = +267 mV, while at pH 7.0, ψ₀ ≈ +31 mV. Based on these values, the optimal pH for adsorption (7.0, near neutrality) reflects a balance: electrostatic effects are minimal near the pHpzc, while hydrophobic interactions between the dodecyl chain of SDS and the graphitic basal planes of anthracite, along with hydrogen bonding between the sulfonate headgroup and surface hydroxyl groups, become dominant. Higher pH, electrostatic repulsion outweighs these attractive forces. At lower pH, although electrostatic attraction increases, the reduced dissociation of SDS (pKa ≈ 1–2 for the sulfate ester) lowers the concentration of anionic species, limiting uptake. Consequently, pH 7 was identified as ideal for SDS uptake by anthracite, yielding a maximum adsorption capacity of 118.9 ± 5 mg/g.

#### Influence of adsorbent dosage

Each sample was submerged in 25 mL of an SDS solution at a concentration of 120 mg/L, kept at 25 °C, and used adsorbent masses ranging from 2 to 40 mg. The system’s pH had stabilized to about 7 before the experiments started, and the stirring rate had been adjusted to its ideal level. A total of 120 min were spent on the adsorption process. The impact of adsorbent dosage on SDS removal efficiency was methodically assessed, as shown in Fig. 6(b). The experimental findings show a clear relationship between the removal efficiency (%R) of SDS and adsorbent mass. In particular, the elimination efficiency increased from 20.83% to 98.25% when the adsorbent dosage increased from 2 mg to 40 mg.

The enhanced availability of active adsorption sites at higher adsorbent concentrations, which promotes greater contaminant uptake, is responsible for this notable improvement in SDS removal^46,47^. Under constant-volume adsorption conditions, the active sites are not fully utilized, which results in unsaturated adsorption capacity. Interestingly, as adsorbent mass rises at a specific SDS concentration, the equilibrium adsorption capacity (q_e_) shows an inverse relationship with removal efficiency (R%). Higher adsorbent loading reduces uptake per unit mass by distributing the fixed contaminant mass over a larger number of active sites. Moreover, particle aggregation may be induced by excessive adsorbent quantities, reducing the effective surface area available for adsorption.

In addition to decreasing interfacial contact, this agglomeration effect lengthens the contaminant molecules’ diffusion pathway, which may jeopardize adsorption kinetics. The mass-normalized adsorption capacity at time *t* (q_e_) and the adsorbent mass (m) are inversely proportional, according to Eq. (2), assuming that the initial SDS concentration (C₀), solution volume (V), and removal efficiency (%R) stay constant. The reason for this relationship is that a larger adsorbent mass reduces the adsorbed amount per unit mass (q_e_) by distributing the fixed SDS quantity over a larger number of active sites. Because the same contaminant load is distributed over more binding sites, increasing the adsorbent dosage reduces its per-gram adsorption efficiency.

The most economical equilibrium is represented by the convergence point of the removal efficiency (R%) and adsorption capacity (q_e_) curves, which should be used to calculate the ideal adsorbent dosage. The system strikes the ideal balance between minimizing resource consumption and optimizing SDS elimination at this crucial point. This strategy preserves operational effectiveness and financial feasibility while avoiding diminishing returns from excessive adsorbent application. Thus, the intersection ensures effective contaminant removal without needless material consumption, making it a strategic benchmark for the design of sustainable adsorption processes^28,42^.

#### Influence of agitation time

As shown in Fig. 6c, a series of time-dependent batch experiments were used to examine the adsorption kinetics of SDS onto An. 50.83% of the surfactant was removed in the first two minutes, demonstrating the system’s quick initial adsorption kinetics. This instant uptake indicates that there were a lot of active adsorption sites present during the first phase. Removal efficiency increased gradually, reaching 64.17% after 5 min and 70.00% after 15 min. With longer contact times, the adsorption rate gradually decreased, reaching 80.00% removal at 30 min and 85.83% after 60 min. With an efficiency of 97.92%, the system achieved near-complete SDS removal at 120 min, approaching equilibrium conditions.

This kinetic profile shows a fast adsorption phase at first, followed by a slower uptake as the number of available adsorption sites dwindled. After 180 min of contact time, the adsorption process reached its maximum efficiency (98.17%), with only a slight improvement observed after 120 min. A distinctive biphasic adsorption mechanism on An is revealed by this kinetic pattern, which consists of a fast surface adsorption phase at first, followed by slower intraparticle diffusion as the system gets closer to saturation. The experimental results show that 120 min is the ideal contact time to reach near-equilibrium conditions, while longer exposure times result in only a slight increase in removal efficiency. These results demonstrate An’s outstanding performance in SDS elimination, which is supported by both its high ultimate removal capacity and quick initial uptake.

The material’s adsorption efficacy and observed kinetic behavior point to a high degree of practical implementation potential in greywater remediation applications where processing time and efficiency are crucial operational parameters^48,49^.

#### Influence of concentration

The impact of the initial surfactant concentration on the adsorption efficiency of anthracite coal is illustrated in Fig. 6d. Throughout the experiment, the following parameters were kept constant: pH 7, temperature 298 K, 20 mg L⁻¹ of adsorbent, and 120 min of contact time. According to the data, anthracite coal exhibited consistently high removal efficiencies within the concentration range of 5–120 mg L⁻¹. The removal efficiencies were exceptionally high, ranging from 99.5% to 99.6% at lower concentrations (5–20 mg L⁻¹). This outstanding performance remained consistent up to 60 mg L⁻¹, with removal efficiencies remaining at 99.5%. At higher concentrations, values dropped slightly to 98.88% and 98.83% at 80 and 120 mg L⁻¹, respectively.

This slight decrease at higher concentrations may be due to the limited number of active adsorption sites on the surface of anthracite coal, which become more saturated as the surfactant loading increases. On the other hand, the consistently high removal efficiency across the whole range show that anthracite coal has a considerable sorption capacity under the tested conditions, suggesting that it could be an effective adsorbent for treating surfactant-contaminated greywater^50^.

### Adsorption isotherms

**Fig. 7:**
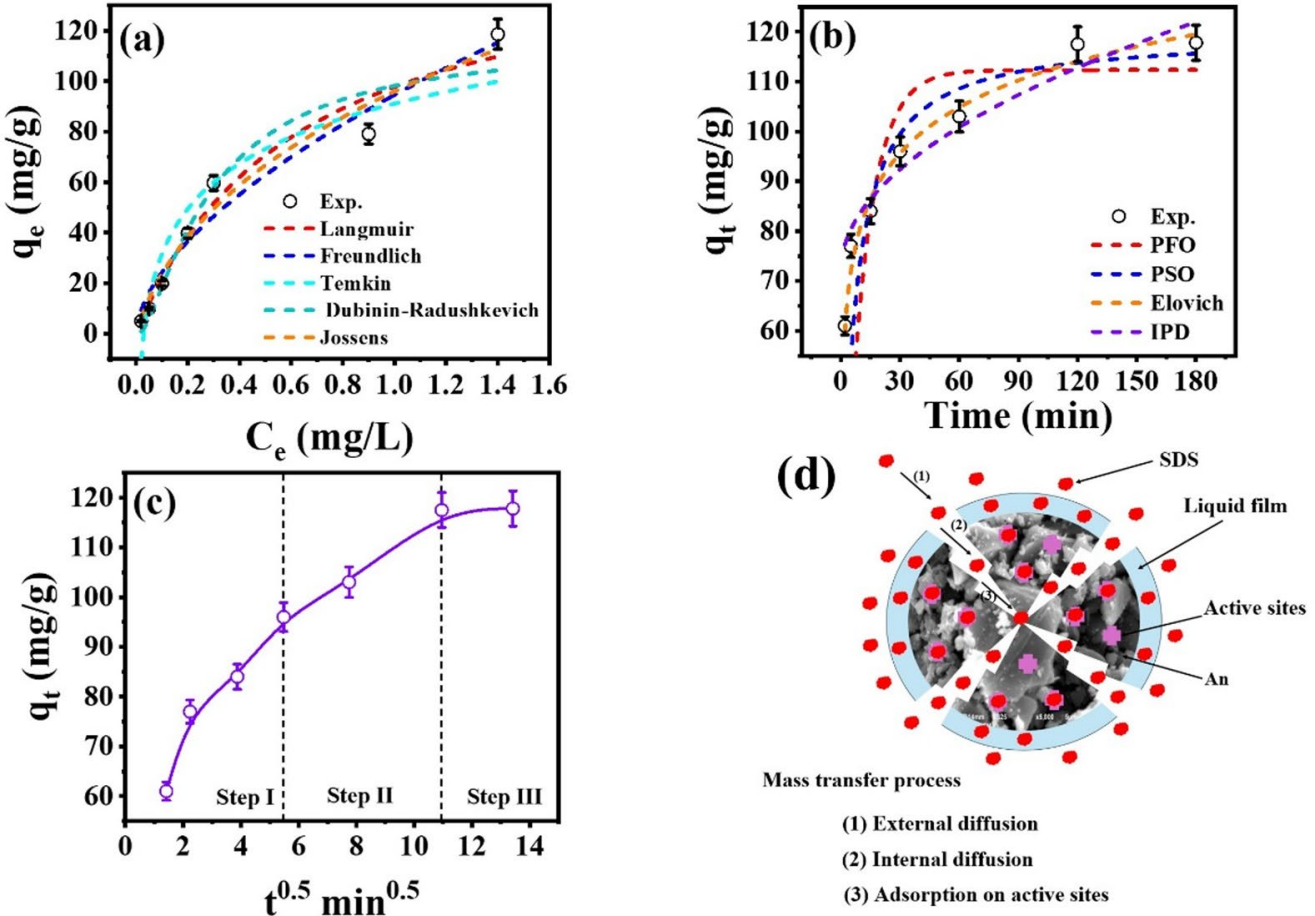
(**a**) Models of the adsorption isotherms, (**b**) Models of the adsorption kinetics, (**c**) IPD model, and (**d**) Representation diagram of diffusion mechanism of SDS ions onto An.

Under optimized experimental conditions, the equilibrium between adsorbate and adsorbent was modeled by fitting the collected data to various isotherm expressions (Table 4). The reliability of each fitted model was evaluated using the coefficient of determination (R²), the chi-squared statistic (X²), and the root mean square error (RMSE); the complete set of derived values is summarized in Table 4. Based on the statistical metrics employed, the Jossens isotherm model (R² = 0.969) emerged as the most accurate descriptor of the adsorption behavior observed in the system. This robust alignment with the Jossens framework offers theoretical support for the presence of an energetically nonuniform surface on the An adsorbent, where adsorption sites exhibit a continuous distribution of binding strengths. The aforementioned surface heterogeneity was quantitatively characterized through the isotherm parameters. Specifically, a substantial equilibrium constant (K = 391.5) signifies a strong thermodynamic impetus for SDS adsorption, whereas the heterogeneity factor (J = 3.08) verifies considerable variation in site energy levels. Taken together, these findings point to a marked capacity for the efficient removal of SDS by the adsorbent^21,39^. The heterogeneity originally attributed to the adsorption mechanism was reevaluated through comparative isotherm modeling. Although the Langmuir and Freundlich equations each yielded a statistically acceptable fit, the Langmuir model demonstrated marginally superior performance relative to the Freundlich framework^51^, as evidenced by a higher R² and lower X² and RMSE values. This finding supports a monolayer adsorption regime occurring on a uniform surface. The Freundlich exponent, 1/n, recorded a value below unity, which further indicates a heterogeneous distribution of bond energies consistent with a favorable chemisorption process^41,52^. A strong alignment with the D-R isotherm^39^ was observed for the experimental dataset (R² = 0.948), suggesting that a pore-filling mechanism adequately describes the adsorption phenomenon. In addition, the Temkin isotherm model, employed to examine SDS uptake onto An, offers distinct theoretical benefits. Notably, its explicit inclusion of adsorbate–adsorbate interactions an aspect routinely neglected in simpler formulations represents a key advantage. Consequently, this approach enables a more nuanced depiction of the adsorption behavior, as the model assumes a linear decline in the adsorption enthalpy with progressive surface coverage. The calculated Temkin parameters specifically, a b_T_ constant of 25.9 kJ/mol and an A_T_ value of 33.6 L/g (as presented in Table 4) indicate a moderate but meaningful energy interaction between SDS molecules and the An adsorbent. This particular range of the b_T_ coefficient holds notable relevance for real-world applications necessitating adsorbent recycling, since it reflects an adsorption affinity that is neither too strong to hinder effective desorption nor too weak to undermine overall uptake performance^18^. Furthermore Fig. 7a illustrates the nonlinear regression of the equilibrium datasets applied to several isotherm models. Of those evaluated, the Jossens model demonstrates the strongest alignment with empirical observations, thereby establishing it as the most suitable representation of the adsorption behavior.

**Table 4:** The parameter of the adsorption isotherm for SDS on An.

Model	Regression	Parameter	Unit	Value	Std. error	t- value	Pr (>|t|)
Langmuir	$\:{\mathrm{q}}_{\mathrm{e}}={\mathrm{q}}_{\mathrm{m}\mathrm{a}\mathrm{x}}\frac{{\mathrm{K}}_{\mathrm{L}}{\mathrm{C}}_{\mathrm{e}}}{1+{\mathrm{K}}_{\mathrm{L}}{\mathrm{C}}_{\mathrm{e}}}$ $\:{\mathrm{R}}_{\mathrm{L}}=\frac{1}{1+{\mathrm{K}}_{\mathrm{L}}{\mathrm{C}}_{\mathrm{o}}}$	q_max_ K_L_ R_L_ R^2^ Adj – R^2^ X^2^ RMSE	mg/g L/mg unitles	158.7 1.6 0.11–0.005 0.96 0.95 6.52 7.25	24.30 0.547	6.529 2.928	0.001 0.032
Freundlich	q_e_ = K_f_ C_e_^1/n^	K_f_ 1/n R^2^ Adj – R^2^ X^2^ RMSE	(mg/g)(L/mg)	94.6 0.59 0.964 0.956 13.25 7.26	5.159 0.205	18.322 8.255	8.903 4.251
Temkin	q_e_ = q_max_ $\:\frac{\mathrm{R}\mathrm{T}}{\mathrm{b}}\mathrm{l}\mathrm{n}({\mathrm{K}}_{\mathrm{T}}{\mathrm{C}}_{\mathrm{e}}$)	A_T_ b_T_ R^2^ Adj – R^2^ X^2^ RMSE	L/g J/mol	33.6 25.9 0.911 0.893 61.29 11.39	10.842 3.618	3.096 7.165	0.026 8.230
D-R	$\:{\mathrm{q}}_{\mathrm{e}}={\mathrm{q}}_{\mathrm{m}\mathrm{a}\mathrm{x}}\mathrm{exp}(-{\upbeta\:}{{\upepsilon\:}}^{2})$ $\:{\upepsilon\:}=\mathrm{R}\mathrm{T}\mathrm{l}\mathrm{n}(1+\frac{1}{{\mathrm{C}}_{\mathrm{e}}})$ $\:{\mathrm{E}}_{\mathrm{D}\mathrm{R}}=\sqrt{\frac{1}{2{\mathrm{K}}_{\mathrm{D}\mathrm{R}}}}$	Q_DR_ K_DR_ E_a_ R^2^ Adj – R^2^ X^2^ RMSE	mg/g mol^2^ /KJ^2^ J/mol	114.302 0.316 1.3 0.948 0.938 10.42 8.65	9.576 0.059	11.936 5.360	7.275 0.003
Jossens	$\:{q}_{e}=\frac{K{\times\:\:C}_{e}}{(1+j\:\times\:\:{C}_{e}^{n})}$	K j n R^2^ Adj – R^2^ X^2^ RMSE		391.5 3.08 0.67 0.969 0.953 5.06 6.71	472.39 5.066 0.456	0.828 0.608 1.472	0.453 0.575 0.214

### Adsorption kinetics

The mechanism underlying SDS adsorption onto the An surface was examined through the application of four kinetic models namely, pseudo-first-order (PFO), pseudo-second-order (PSO), Elovich, and intraparticle diffusion (IPD)^21,39,53,54^. This integrative analytical framework not only delineates the prevailing process dynamics but also furnishes a rigorous empirical foundation for optimizing system parameters to enhance pollutant removal efficiency. Nonlinear regression analysis of the time-dependent adsorption capacity (q_t_) derived from SDS adsorption experiments (Fig. 7b), performed against the PFO and PSO kinetic models, produced the parameters summarized in Table 5. The statistical outcomes unequivocally demonstrate that the PSO model outperforms the PFO model in describing the adsorption process. This superiority is evidenced by a R² = 0.836 for PSO versus 0.639 for PFO and consistently lower error function values. A key corroboration of this observation lies in the minimal discrepancy between the equilibrium uptake capacities (q_e_) derived from the PSO model and those obtained experimentally. The PSO model’s strong adherence to the kinetic data confirms its suitability as a reliable representation of SDS adsorption. This outcome substantiates the inference that chemisorption constitutes the predominant mechanism governing the removal process at the adsorbent interface. The relatively low value of the PSO rate constant (K₂ = 0.0044 g·mg⁻¹·min⁻¹) further indicates a slow but efficient adsorption mechanism, one that remains capable of delivering significant pollutant elimination over prolonged durations of system operation^53,55^.

A robust correlation with the Elovich model (R² = 0.984) substantiates the existence of a heterogeneous surface morphology on the adsorbent material, a defining feature depicted in Fig. 7b. The associated kinetic parameters namely, the initial adsorption rate (α = 0.079 mg·g⁻¹·min⁻¹) and the surface coverage-related energy coefficient (β = 818.1 g/mg) provide a quantitative characterization of the adsorption dynamics. These values capture both the swift early-stage uptake and the progressive reduction in adsorption energy as active sites become increasingly occupied. The kinetic evaluation uncovers two essential characteristics: the magnitude of α indicates sustained adsorptive capacity over extended durations, whereas the notably high β parameter corroborates a sharp rise in activation energy as surface sites become progressively occupied. Such a kinetic signature typical of chemisorption on heterogeneous surfaces not only supports the validity of the pseudo-second-order model but also offers a mechanistic rationale for the enhanced SDS removal performance exhibited by the An material within remediation systems^21^. Employing the Elovich model yields actionable understanding for the treatment of SDS-laden streams with fluctuating concentrations, as it enables the quantification of realistic adsorption kinetics. This analytical strategy elucidates the fundamental interfacial interactions occurring between the An surface and SDS molecules, thus facilitating the design of optimized remediation strategies for recalcitrant dye-containing wastewater. Consequently, its relevance and applicability within the domain of practical environmental science are firmly established^39^.

**Table 5:** Kinetic model fitting parameters for SDS adsorption onto An.

Model	Regression			variable		Unit		Value		Std. error		t- value		Pr (>|t|)	
PFO	q_t_= q_e_ (1-e^k^_1_^t^)			q_e_ K_1_ R^2^ Adj– R^2^		mg/g min^− 1^		103.3 0.35 0.639 0.566		6.241 0.11		16.55 3.13		1.47191E-5 0.026	
PSO	q_t_ = $\:\frac{\mathrm{t}{\mathrm{k}}_{2}{\mathrm{q}}_{\mathrm{e}}^{2}}{1+{\mathrm{q}}_{\mathrm{e}}{\mathrm{k}}_{2}\mathrm{t}}$			q_e_ K_2_ R^2^ Adj– R^2^		mg/g g.mg^− 1^.min^− 1^		110.1 0.0044 0.836 0.804		5.08 0.0015		21.66 2.99		3.88994E-6 0.031	
Elovich	q_t_ = $\:\frac{1}{{\upbeta\:}}\:\mathrm{L}\mathrm{n}({\upalpha\:}{\upbeta\:}\mathrm{t}+1)$			αβ R^2^ Adj– R^2^		mg·g^− 1^·min^− 1^ g/mg		0.079 818.1 0.984 0.980		0.0046 314.25		17.07 2.603		1.26103E-5 0.048	
IPD	q_t_ = K_p_ t^0.5^+C			K_P_ C R^2^ Adj– R^2^		mg·g^− 1^·min^1/2^ mg/g		4.5 64.8 0.914 0.897		0.617 4.737		7.281 13.673		7.64311E-4 3.75206E-5	

Linearized Interparticle diffusion model															
Step 1					Step2						Step3				
k_P_1		**C**_**1**_	R^**2**^		k_**P**_**2**		C_**2**_		R^**2**^		k_**P**_**3**		C_**3**_		R^**2**^
8.6		52.4	0.840		4.8		67.1		0.939		2.7		83.8		0.826

The dominant mass transfer mechanism regulating SDS adsorption onto the An surface was elucidated using the linearized IPD model. As summarized in Table 5, the estimated kinetic parameters (k_p_ and C), together with the linear regression of q_t_ plotted against t⁰·⁵ (Fig. 7c), collectively delineate a three-phase adsorption process. This multiphasic kinetic profile indicates that SDS removal proceeds via complex interfacial dynamics governed by sequential transport resistances. The adsorption process commences with the rapid migration of SDS ions from the bulk solution to the external surface of the An adsorbent a film diffusion-controlled step represented by the initial linear segment of the kinetic curve, which exhibits a positive intercept on the y-axis. The initial phase proceeds at a rate of 8.6 mg·g⁻¹·min⁻¹/². A subsequent, clearly linear segment signals a mechanistic transition toward gradual intraparticle diffusion, during which SDS ions migrate into the internal mesopores and micropores of the material. The restricted diffusion pathways inherent to this porous structure impose considerable mass transfer constraints, as reflected by a markedly lower rate constant of 4.8 mg·g⁻¹·min⁻¹/² a substantial decrease from the initial film diffusion stage (8.6 mg·g⁻¹·min⁻¹/²). A further, distinct linear regime then emerges, operating at a rate of 2.7 mg·g⁻¹·min⁻¹/², which is subsequently identified as the definitive rate-controlling step. The multilinear nature of the kinetic plot offers definitive proof of a two-mechanism adsorption pathway, involving both film diffusion and intraparticle diffusion. Final equilibrium was attained due to the progressive saturation of binding sites on the An surface, coupled with a minimal residual concentration gradient that no longer supplied adequate thermodynamic driving force to sustain further adsorption, as illustrated in Fig. 7c and d^54^.

### Adsorption thermodynamics

**Fig. 8:**
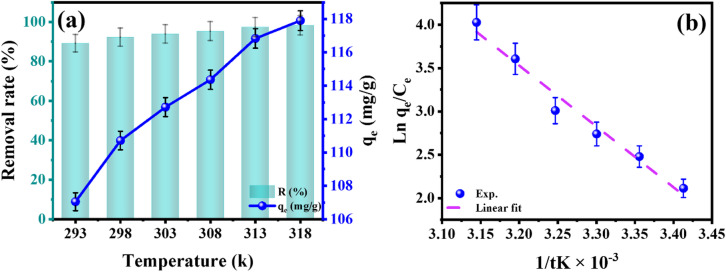
(**a**) Effect of temperature onto adsorption of SDS and (**b**) Van’t Hoff model.

To determine how temperature affected the surfactant’s adsorption efficiency from biologically pretreated greywater using anthracite coal, several batch experiments were carried out at different temperatures between 293 and 318 K. As Fig. 8a illustrates, the removal efficiency rose significantly as the temperature rose. The removal efficiency at 293 K was only 89.2%, which was comparatively moderately low. However, efficiency improved noticeably at 298 K, reaching 92.2%. As the temperature increased to 303, 308, and 313 K, respectively, performance improved even more; efficiencies of 93.9%, 95.3%, and 97.35% were noted. At 318 K, the highest removal efficiency was 98.25%. The surfactant molecules’ enhanced kinetic energy and better molecular diffusion are responsible for the slow increase in adsorption efficiency. These factors probably promoted stronger interactions with the active adsorption sites on the anthracite coal surface^50^.

**Table 6:** Thermodynamic parameters for the adsorption of SDS onto An.

Adsorbent	ΔH^o^ (kJ/mol)	ΔS^o^ (J/molK)	ΔG^o^ (kJ/mol)						*R* ^2^
			293 k	298 k	303 k	308 k	313 k	318 k	
Anthracite coal	58.4	216.2	-5.15	-6.14	-6.91	-7.71	-9.38	-10.65	0.974

Table 6 illustrates the thermodynamic parameters that described the detergent’s adsorption onto anthracite coal and indicated a highly favorable process. The standard Gibbs free energy change (ΔG°) values ranged from − 5.15 to − 10.65 kJ mol⁻¹ over the temperature range of 293 to 318 K, suggesting a notable increase in spontaneity with temperature.

This notable drop in ΔG° suggests that as temperatures rise, the adsorption process becomes more thermodynamically beneficial. The positive enthalpy change (ΔH° = 58.4 kJ mol⁻¹) indicates an endothermic nature, indicating that thermal input raises the affinity between the detergent molecules and the anthracite coal surface^50^.

Furthermore, strong interactions that might involve chemisorption mechanisms are indicated by a high ΔH° value. Furthermore, a significant increase in disorder at the solid–liquid interface is indicated by the large positive entropy change (ΔS° = 216.2 J mol⁻¹ K⁻¹), which could be caused by the solvent molecules being released during detergent adsorption. The notion that increased randomness and system disarray accompany adsorption is supported by this entropy gain. Together, these results demonstrate that the detergent uptake onto anthracite coal happens via an endothermic, spontaneous process that significantly increases disorder. This is consistent with adsorption that involves strong surface interactions and substantial structural rearrangement^39^.

### Comparative study

A systematic comparison presented in Table 7 evaluates the adsorption performance of the An material against previously reported adsorbents for SDS removal under different operational conditions, including pH, adsorbent dosage, and contact time. The An sorbent exhibited a maximum adsorption capacity (q_m_) of 158.692 mg/g, demonstrating superior adsorption efficiency compared with several conventional adsorbents reported in the literature, such as modified cellulose with quaternary ammonium (32.5 mg/g), activated coconut shell carbon (28.57 mg/g), modified natural zeolite (30.70 mg/g), and chitosan hydrogel beads (76.9 mg/g). Although certain adsorbents, including tannin (960 mg/g), activated carbon with bacteria (468.8 mg/g), and ZIF-8/carbon fiber (377 mg/g), exhibited comparatively higher adsorption capacities, the An material demonstrated remarkable adsorption performance under relatively moderate operational conditions (pH 7, 120 min, and 20 mg/25 mL dosage). These findings highlight the strong affinity of the An toward SDS molecules and confirm its potential applicability as an efficient and promising adsorbent for surfactant-contaminated wastewater treatment.

**Table 7:** Maximum adsorption capacities of multiple composites towards the removal of SDS in previous published work.

Adsorbents	Ideal conditions			Qm (mg/g)	Ref.
	pH	dose	t (min)		
Modified Cellulose with Quaternary Ammonium	7.0	-	180	32.5	^56^
ZIF-8/carbon fiber	5.0	1 g/L	30	377	^57^
activated coconut shell carbon	2.0	3 g/L	180	28.57	^58^
Modified natural zeolite	9.0	2.5 g/L	180	30.70	^59^
chitosan hydrogel beads	-	0.5 g/L	180	76.9	^51^
Activated carbon with bacteria	7.0	1 g/L	180	468.8	^60^
Tannin	4.9	0.05 g/L	120	960	^61^
Coal	5.5	5 g/L	-	30	^62^
An	7.0	20 mg/25 ml	120	158.692	This study

### Adsorption mechanism

**Fig. 9:**
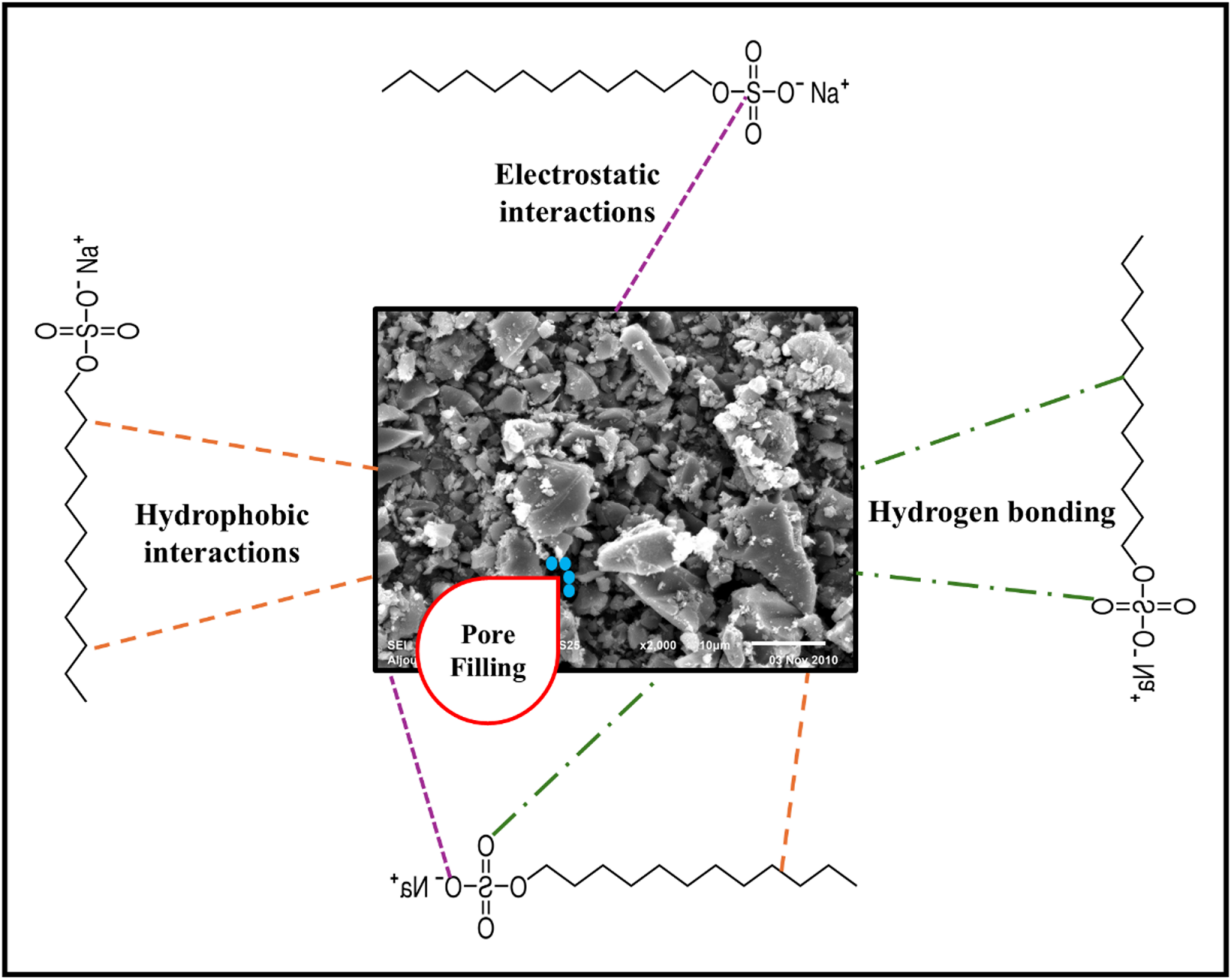
Reaction mechanism for SDS adsorption on An.

The adsorption of SDS onto An occurred through a cooperative multi-mechanistic pathway involving intra-particle pore diffusion, hydrophobic attraction, electrostatic interactions, and hydrogen bonding (Fig. 9). Kinetic analysis using the IPD model revealed three successive adsorption stages corresponding to external mass transfer, mesopore diffusion, and micropore diffusion, confirming that pore diffusion significantly contributed to SDS uptake. This behavior was supported by the high BET surface area (890.9 m² g⁻¹), micro–mesoporous structure, and suitable pore diameter (2.49 nm) of An, which facilitated the transport and accommodation of SDS molecules within the pore network. Thermodynamic parameters further demonstrated that adsorption was spontaneous and endothermic, while the large positive entropy value indicated strong hydrophobic interactions between the non-polar dodecyl chains of SDS and the graphitic domains of anthracite, particularly at elevated temperatures.

The adsorption process was also strongly affected by solution pH, with maximum adsorption occurring near neutral conditions close to the pHpzc value (7.52), indicating the important role of electrostatic interactions between the negatively charged SDS headgroups and the An surface functional groups. FTIR analysis confirmed the participation of hydroxyl and oxygen-containing groups in hydrogen bonding interactions with the sulfonate headgroups of SDS. In addition, the adsorption isotherm behavior and the superior fitting of heterogeneous adsorption models suggested cooperative adsorption and hemi-micellar aggregation on the anthracite surface at high SDS loading. The high adsorption capacity together with the positive entropy change further supported the formation of surface aggregates and multilayer adsorption driven by combined hydrophobic and interfacial interactions.

###  Effect of interference substances

**Fig. 10:**
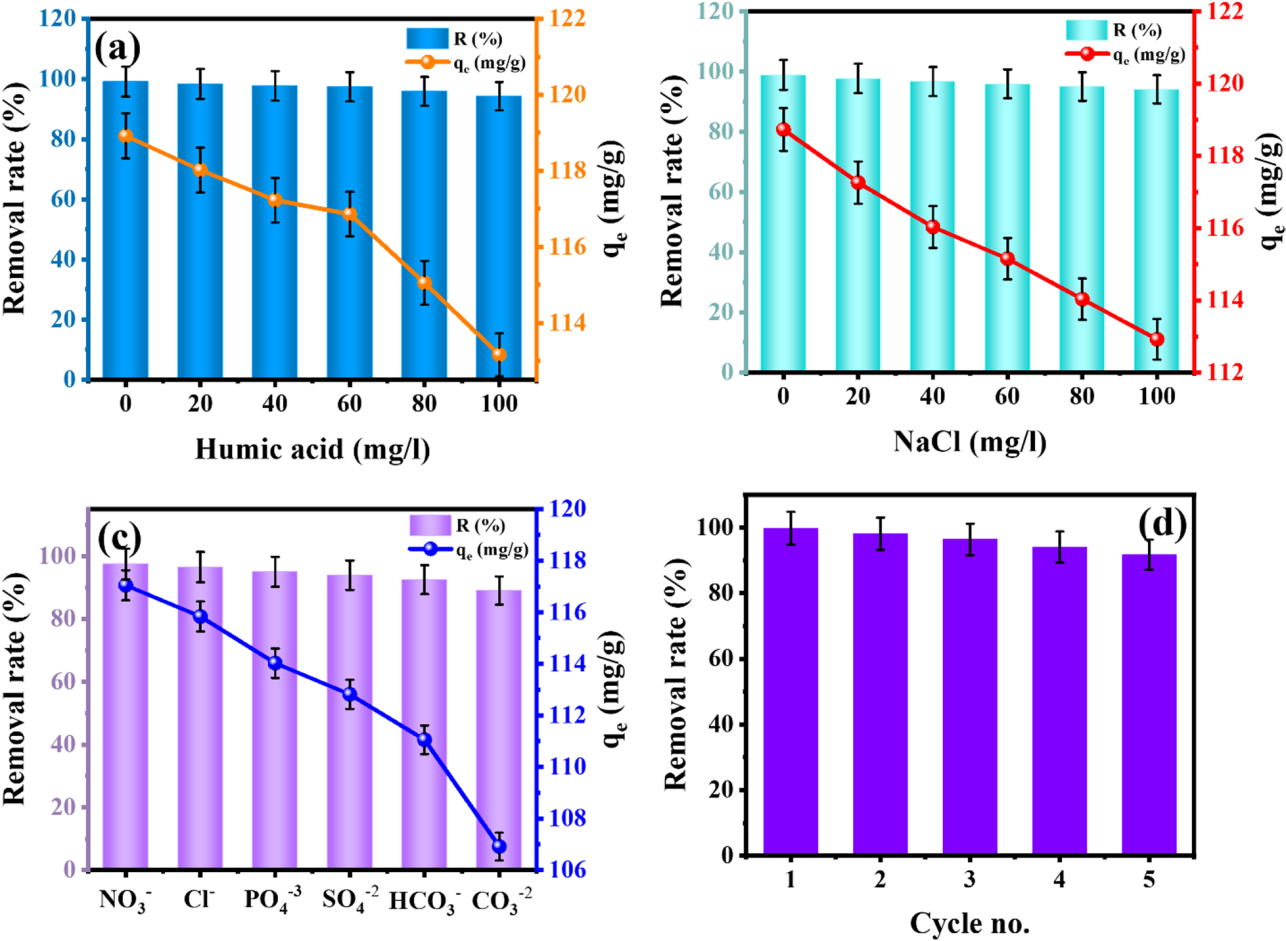
Influence of (**a**) humic acid concentration, (**b**) ionic strength, (**c**) coexisting ionic species, and (**d**) cyclical regeneration on the adsorptive performance of An.

To evaluate the risk of interference caused by competing species, the widespread occurrence of natural organic matter (NOM) in aqueous environments was taken into account. A comparative adsorption experiment was performed by incorporating humic acid (HA) employed as a representative NOM substance into SDS solutions at concentrations ranging from 0 to 100 mg/L. This approach aimed to determine the extent to which HA could occupy binding sites on the An adsorbent, thereby reducing its capacity for SDS uptake. Even at the highest NOM concentration of 100 mg/L, the An adsorbent sustained an SDS removal efficiency exceeding 94.3%, although a modest decline in q_e_ values was observed with rising HA levels (Fig. 10a). Such resilience in the face of adverse conditions underscores the material’s superior adsorptive capacity as well as its resilience against interference from organic substances^63^.

Empirical findings indicate that ionic strength plays a decisive role in governing the adsorption process. An examination of the influence exerted by varying sodium chloride concentrations demonstrated a negative correlation with adsorptive performance; specifically, the qₑ value of An declined from 118.73 mg/g to 112.92 mg/g as the NaCl concentration rose from 0 to 100 mg/L (Fig. 10b). The reduction in An’s adsorptive capacity observed under elevated ionic strength conditions points to a charge-shielding mechanism driven by anions^64^. To explore this anion-dependent phenomenon, a comparative analysis was performed using solutions containing NaCl, Na₂SO₄, NaNO₃, NaHCO₃, and Na₂CO₃, each maintained at a concentration of 50 mg/L. The outcomes conclusively establish CO₃²⁻ and HCO₃⁻ as the strongest suppressors of adsorption (Fig. 10c), suggesting that these anions possess superior shielding abilities relative to other anionic species. The predominance of carbonate and bicarbonate anions may be attributed to a dual mechanism. Firstly, the high charge density of CO₃²⁻ enables it to vie effectively for the protonated active sites on the adsorbent. Secondly, proton consumption resulting from the hydrolysis of these anions elevates the pH of the surrounding solution. This rise in pH subsequently diminishes the positive surface charge on the adsorbent, thereby severely impairing the electrostatic mechanism responsible for SDS uptake^21,23^.

### Reusability test

To assess the chemical robustness and operational efficacy of the An adsorbent, a series of reusability trials were undertaken involving five consecutive adsorption–desorption cycles for the elimination of SDS from aqueous solutions. Following the initial regeneration step, the SDS removal efficiency (R%) reached 99.6%, which thereafter declined to 91.68% after the fifth cycle (Fig. 10d), employing 1 M HCl as the washing agent. This progressive reduction in performance is attributed to potential degradation or leaching of the active functional groups resulting from repeated exposure to acidic regeneration conditions. Based on these observations, it is concluded that the An adsorbent demonstrates substantial reusability, retaining high SDS removal capacity from aqueous media across multiple operational cycles with no substantial loss in performance^43,50^.

### Synergistic interplay between biodegradation and adsorption processes

The hybrid treatment system developed in this study demonstrated superior remediation performance due to the strong synergistic integration between biological degradation and adsorption processes rather than the independent efficiency of each treatment stage. The biodegradation step, conducted under optimized conditions of pH 7.9 and 25 °C, achieved 90.46% removal of sodium dodecyl sulfate (SDS), leading to the transformation of the parent surfactant molecules into smaller and less complex intermediates such as fatty acids, sulfates, and alcohols. This partial mineralization substantially reduced the organic load and minimized the foaming behavior associated with SDS, which could otherwise hinder adsorption efficiency through pore blockage and competitive occupation of active sites. Consequently, the adsorption stage received a chemically modified effluent containing only residual SDS and low-molecular-weight degradation products, thereby improving the accessibility of adsorption sites and enhancing the polishing efficiency of the downstream process.

The biological pretreatment additionally established favorable physicochemical conditions for adsorption. The near-neutral pH of the biodegraded effluent (pH 7.9) closely matched the optimum adsorption pH (7.0), thus requiring minimal pH adjustment prior to the polishing step. This compatibility maintained the anthracite surface near its point of zero charge (pHpzc = 7.52), promoting balanced electrostatic interactions with residual anionic SDS species as well as positively charged metabolic intermediates. Such conditions facilitated more effective adsorption through a combination of electrostatic attraction, hydrogen bonding, and surface interactions.

The sequential coupling of biodegradation and adsorption also generated a complementary removal mechanism capable of overcoming the limitations associated with each standalone process. Biological treatment efficiently handled the high initial SDS concentration (300 mg L⁻¹), thereby preventing the premature saturation of adsorption sites that would occur if adsorption were applied directly. Subsequently, the adsorption stage acted as an effective polishing unit, increasing the overall removal efficiency to 99.58%. This integrated configuration therefore combined the high contaminant-handling capacity of biodegradation with the high finishing efficiency of adsorption, enabling near-complete remediation within practical operational conditions.

Moreover, the metabolic activity of *Serratia plymuthica* BSU-AH-03 during the biodegradation process likely contributed to additional enhancement of adsorption performance through the production of extracellular polymeric substances (EPS) and biosurfactants. These biologically derived compounds may condition the anthracite surface by introducing supplementary oxygen- and nitrogen-containing functional groups, including carboxyl, phosphoryl, and amine moieties. The presence of such functionalities potentially created additional binding domains capable of interacting with residual SDS molecules through hydrogen bonding, ion–dipole interactions, and surface complexation mechanisms beyond those originally available on the raw anthracite surface.

The thermodynamic compatibility between the two treatment stages further strengthened the efficiency of the integrated system. While biodegradation exhibited optimal activity within the mesophilic temperature range around 25 °C, the adsorption process demonstrated increasing spontaneity with rising temperature, as evidenced by the progressive decrease in ΔG° values from (ΔG° from – -5.15 to -10.65 kJ mol⁻¹) over the temperature range of 293–318 K. The metabolic heat generated during microbial activity within the bioreactor may therefore contribute to enhancing adsorption kinetics in the subsequent polishing stage without requiring additional external energy input, thereby improving the overall energy efficiency and sustainability of the hybrid treatment system.

## Conclusion

This study successfully developed an efficient hybrid treatment system for the remediation of sodium dodecyl sulfate (SDS)-contaminated greywater through the integration of optimized biological degradation and anthracite-based adsorption. A novel SDS-degrading bacterial strain, *Serratia plymuthica BSU-AH-03*, was isolated and statistically optimized using Box–Behnken Design under Response Surface Methodology, achieving a biodegradation efficiency of 90.46% under the optimum conditions of pH 7.9, 20 °C, and 300 mg L⁻¹ SDS, with excellent model predictability (R² = 0.981). The natural anthracite adsorbent exhibited a high BET surface area (890.9 m² g⁻¹), heterogeneous micro–mesoporous structure, and thermally stable functional groups, resulting in a maximum adsorption capacity of 158.7 mg g⁻¹ and an overall SDS removal efficiency of 99.58% under optimized adsorption conditions. Comparative evaluation confirmed that the adsorption performance of anthracite was superior or comparable to many previously reported adsorbents for SDS removal.

Biodegradation peaked near 25 °C (mesophilic range), whereas adsorption became more spontaneous with rising temperature. Over 293–318 K, ΔG° values decreased from − 5.15 to − 10.65 kJ mol⁻¹, confirming enhanced thermodynamic favorability for SDS removal at higher temperatures. Thermodynamic and kinetic analyses demonstrated that the adsorption process was spontaneous and endothermic, while the fitting of the Langmuir isotherm and pseudo-second-order kinetic models indicated the dominant role of surface interactions during SDS uptake. Mechanistic investigation revealed that SDS sequestration occurred through multiple pathways, including pore diffusion, hydrophobic attraction, electrostatic interactions, hydrogen bonding, and hemi-micellar surface assembly. The sequential integration of biodegradation and adsorption effectively overcame the limitations of the individual processes, where biodegradation reduced the initial surfactant load and adsorption acted as an efficient polishing stage. Owing to its high efficiency, low-cost materials, and operational simplicity, the developed hybrid system represents a promising and sustainable approach for treating surfactant-rich industrial effluents. Future investigations should focus on continuous-flow applications, adsorbent regeneration, and validation using real industrial wastewater systems.

## Data Availability

All data generated or analyzed during this study are included in this published article and its Supplementary Information files. Additional information is available from the corresponding author upon reasonable request.
